# Study of the Relationship between the Structural Parameters of Magnetic Polypropylene-Knitted Fabric and Human Skin Microcirculation

**DOI:** 10.3390/ma14164368

**Published:** 2021-08-04

**Authors:** Zimin Jin, Si Chen, Jing Jin, Kunying Chen, Yuqiang Sun, Mingtao Zhao

**Affiliations:** 1 College of Textile Science and Engineering, Zhejiang Sci-Tech University, Hangzhou 310018, China; icecilechen@163.com (S.C.); cky9128378892021@163.com (K.C.); 2 Animation & Game College, Hangzhou Vocational & Technical College, Hangzhou 310018, China; sky_0120123@163.com; 3 College of Life Sciences and Medicine, Zhejiang Sci-Tech University, Hangzhou 310023, China; sunyuqiang@zstu.edu.cn; 4 Zhejiang Bangjie Holding Group Co., Ltd., Yiwu 321017, China; mtzhao@bangjie.cn

**Keywords:** magnetic polypropylene, human skin microcirculation, knitted fabric structural parameters

## Abstract

In this paper, the effects of the structural parameters of magnetic-knitted fabric on human skin microcirculation (HSM) were studied in relation to magnetic polypropylene yarn, which was used as raw material. Three experimental factors were designed: the magnetic powder content of polypropylene, the feeding ratio of magnetic polypropylene (MP) and graphene viscous (GV), and stitch. Twelve pieces of seamless knitted fabric were prepared according to the comprehensive experimental design method. The BIV angiography was used to proceed with an HSM test of about 12 pieces of seamless knitted fabric. The results show the following: The magnetic powder content of polypropylene has the greatest influence on the blood flow promotion multiples of skin–blood microcirculation, followed by the feeding ratio of MP and GV, while stitch has the least influence. When the plating yarn feeding ratio was 100:0, the magnetic powder content of polypropylene was 50%, and the stitch was 1 + 1 false rib, meaning that the fabric could promote HSM more efficiently. This is compared against the common polypropylene knitted fabric, where the blood flow promotion multiples increased by 9.87%. The purpose of this study was to explore the structural parameters of magnetic polypropylene-knitted fabric that has a better effect on promoting HSM, and to provide a reference for the development of functional health-knitted fabrics.

## 1. Introduction

With the development of the social economy and the continuous innovation of science and technology, people are increasingly exposed to more electronic products that produce substantial electromagnetic radiation [1]. There is a steady-state weak magnetic field on the earth’s surface. Many human-made equipment and instruments also have magnetic fields, which are called artificial magnetic fields [2,3]. Due to frequent contact with these artificial magnetic fields, people are increasingly concerned about whether artificial magnetic fields will cause harm to human health. The external magnetic field may cause harm to the human body; however, it can also be used for recuperating the human body through auxiliary treatment [4,5]. Studies have shown that magnetic fields can reduce vascular permeability, promote cell metabolism, blood circulation, subsidence of inflammation, and enhancement of human immune function [6,7]. By applying a certain degree of magnetic field, the local blood microcirculation of the human body can be improved, while metabolic substances can be quickly removed [8,9].

Microcirculation mainly plays the role of material exchange between tissues and blood microcirculation that exists between the arterioles and venules of microvessels [10,11]. The disorder of microcirculation can be divided into structural disorder and functional disorder. A decrease in micro vessel density and the thickening of vessel walls are structural disorders, while a decrease in blood perfusion or the absence of blood perfusion is considered a functional disorder [12]. Skin microcirculation consists of superficial vascular plexus and deep vascular plexus, and blood perfusion is an important test index in the detection of skin microcirculation. As the skin micro vessels are in a dynamic state of diastolic contraction, it can be found that, when biological organisms are in different environments under different stimuli and different states, the blood perfusion volume is different, and it presents dynamic changes through detection, which can reflect skin microcirculation function [13,14,15]. Relevant studies have shown that microcirculation can be effectively improved by increasing blood perfusion, which has also been proved in animal models [16]. Blood flow in important organs of the human body can also be reflected by observing relevant indexes of blood perfusion in human skin tissue [17]. When a magnetic fiber is applied to clothing fabric, the surface magnetic field of the clothing fabric can maintain a weak magnetic field that is needed by the human body, which could accelerate human blood circulation and cell metabolism function, both of which play a role in health care, as well as serving other purposes [18,19].

Magnetic fibers are divided into magnetic non-textile and magnetic textile fibers. At present, the preparation methods of magnetic fiber include the fiber surface coating method, fiber physical and chemical modification, the blending spin method, the 3D printing method, the impregnation method, the position synthesis method, and the electroplating method [20]. Among these, the blending spin method is the most popular method for preparing magnetic fibers. The magnetic powder added to the magnetic fiber is usually a permanent magnet particle. The permanent magnet is a constant magnetic field with no charge and, after the permanent magnet particle in the magnetized magnetic fiber is permanently magnetized, the magnetic fiber will have a persistent magnetic field [21]. The greater the permanent magnet particle content of the magnetic fiber, the stronger the magnetic induction strength of the magnetic fiber; the same is true for the magnetic property of the magnetic fabric. Additionally, only when the surface magnetism of the magnetic fabric is within a certain strength range, can it provide better therapeutic and health care effect [22].

In 2004, Qi et al. [23] made a magnetic fiber by the blending spinning method. Their test showed that the mechanical properties of the fiber decreased with the increase in magnetic powder content. The fiber could improve blood viscosity and promote microcirculation. In Li. [24], the nylon based magnetic fiber was prepared through the blending spinning method, and a weft knitting machine was used to weave a magnetic collar in 2020. The test result showed that, as the magnetic powder content increased, the magnetic induction intensity increased, and the subjects’ blood flow, blood velocity, and body surface temperature increased. These studies have proved that magnetic fibers have a certain effect on human microcirculation.

In this study, magnetic polypropylene (MP) yarn and graphene viscous (GV) yarn were used to design a four-level face yarn ratio and a three-level magnetic powder content of polypropylene and stitch. The 12 seamless knitted fabric samples were designed and woven according to the comprehensive experimental design method. The influences of various factors on human skin microcirculation (HSM) were analyzed by an orthogonal experiment, and the horizontal combination of process parameters with better performance was obtained, providing a reference value for the development of magnetic health care seamless knitting products that are suitable for winter, and thus greatly promoting HSM and skin–blood flow velocity.

## 2. Materials and Methods

### 2.1. The Sample Material

Considering that magnetic strength is directly related to magnetic powder content, polypropylene yarn was purchased directly from Beijing Baiquan Chemical Fiber Factory (Beijing, China); MP fiber was prepared by the blending spin method. Considering the poor moisture absorption of the polypropylene fibers, the production cost, and the heat retention, as well as the antistatic behavior of winter clothing, GV yarn was selected. Meanwhile, Nylon/spandex-coated yarn with better elasticity was chosen. MP yarn and GV yarn were used as raw materials for face yarn and the nylon/spandex coated yarn [22.2dtex(20D) nylon yarn /77.8dtex(70D) spandex yarn] were used as raw materials for lining yarn. The raw materials and specifications of face yarn are shown in Table 1.

### 2.2. Establishment and Preparation of Sample Scheme

This paper studies the effects of three factors on magnetic powder content: polypropylene, the feeding ratio of MP and GV, and stitch. The sample scheme was designed by an orthogonal experiment. The specific factor levels are shown in Table 2.

This paper designed a 4-level face yarn ratio and a 3-level magnetic powder content of polypropylene and stitch. The sample was knitted with an SM8-TOP2S seamless knitting machine and 8-way yarn feeding. The MP and GV were reasonably set at a feeding ratio of: 8-way MP+0-way GV, 100:0; 6-way MP+2-way GV, 75:25; 4-way MP+4-way GV, 50:50; 2-way MP+6-way GV, 25:75. Three kinds of stitches were selected: the weft plain stitch weave with good elongation in vertical and horizontal direction in seamless knitting; 1 + 1 false rib; and 1 + 3 false rib, which enabled the fabric to be warmer and thicker. In this paper, the orthogonal experimental design of the partial addition method was selected to carry out a comprehensive experiment on a 4-level face yarn ratio, a 3-level magnetic powder content of polypropylene, and a stitch, totaling 12 groups of samples. The weaving scheme of the sample fabric is shown in Table 3.

### 2.3. Establishment of a Method for Measuring Microcirculation Blood Perfusion in Skin

Laboratory equipment: The BVI Angiography provided by Shenzhen Shengqiang Technology Co., Ltd. (Shenzhen, China).

The blood flow imaging function of the BVI Angiography instrument can be used to test the living tissue with an 18 cm × 12 cm test area, and the change in blood perfusion in living tissue can be observed in real time. It is suitable for surgical wound evaluation, cerebral blood flow detection, burn recovery diagnosis, and other fields.

The BVI Angiography instrument’s working principle is as follows: Use the principle of laser speckle imaging when the host sends a laser to the surface of the object. Due to the roughness of the tissue surface, the incident light produced backscatter and random interference upon the imaging surface, thus forming speckle with varying degrees of light and dark. After the image processing software system connected to the computer by the BVI angiography device is converted, we saw the image of the blood vessels on the tissue surface, and obtained the value of the blood perfusion of the blood vessels on the tissue’s surface. The data obtained from the test cannot directly obtain the exact value of blood flow, but they can directly reflect the change in blood flow perfusion volume of the tested tissue.

Experimental environment: Ambient temperature is 24~26 °C, relative humidity is 40~60%, indoor lighting, no wind, avoid direct sunlight.

Experimental requirements: Before the experiment, the sample was put in a standard atmospheric environment for testing (damp) for 24 h. Two test points were marked for subjects in advance. The test points were kept at the same position to avoid any influence on the test results. Subjects were not allowed to consume strong tea, coffee, milk tea, or food containing polyphenols 2 h before the formal test. They sat in the test environment for more than 30 min to avoid the influence of biological physiological changes on the test results. After the subject relaxed, the non-dominant hand was placed on the experimental platform and covered with cotton cloth for more than 15 min to avoid changes in the blood microcirculation of the biological body caused by exercise, which may affect the test results. During the test, the subject’s left arm should be kept quiet in the laboratory, and the subject’s left arm should be relaxed and kept still. The sitting position should not cause any pressure to be applied on the left arm. After obtaining the blood perfusion, the subject’s left arm could move slightly to avoid paralysis of the left arm caused by being placed on the experimental platform for an extended duration.

Subject condition: To avoid the influence of individual differences on the test results, ten healthy female adults, aged 22–26, 155–165 cm in height, and 45–70 kg in weight, without diabetes, somatosensory disorders, hypertension, cardiovascular diseases, and other diseases, were selected [14].

**Figure 1:**
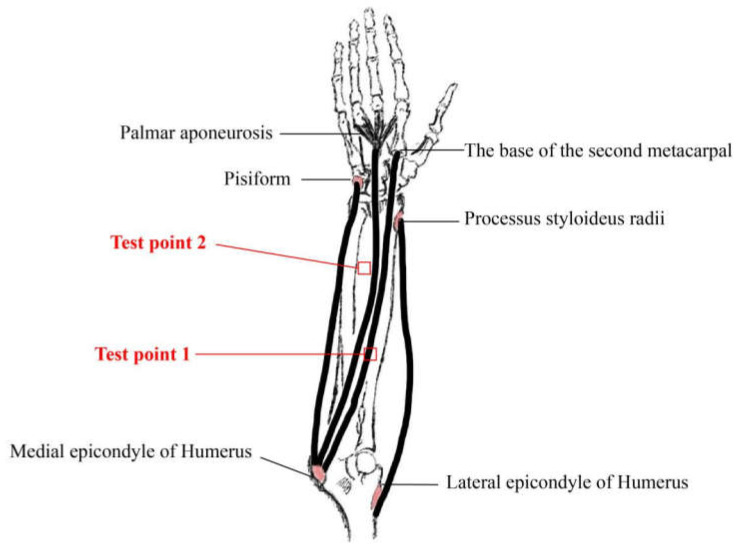
Location of skin–blood microcirculation test points.

Skin microcirculation test position: Combined with the feasibility of the experiment, the selected test points should avoid superficial veins visible on the skin’s surface of the human body; the distance from the wrist was no less than 5 cm and the distance from the elbow fossa was no less than 2.5 cm. The test points with a skin area of about 1.5 cm^2^ were selected, while the test position and test points were the forearm of the subjects’ non-dominant hand, as shown in Figure 1.

Establishment of fabric sample test scheme: Determination of test scheme of fabric-perforated, width, and covered time.

Sample selection: MP-10 Weft plain stitch knitted fabric was used at test point 1 and MP-0 Weft plain stitch knitted fabric was used at test point 2.

In order to make the test results more accurate and reduce the influence of the test results, three factors were considered: the fabric-perforated test scheme, the fabric width, and the fabric covered time. Three perforated test schemes were designed: (1) the sample did not perforate; (2) the sample was not covered or perforated; (3) The sample was perforated and covered. When covering the sample, the test point covered the round sample with a diameter of 2.2 cm. Four different coverage sample widths were designed: 6 cm, 8 cm, 10 cm, and 12 cm. Five different sample covered time schemes were designed: 5 min, 10 min, 15 min, 20 min, and 25 min. According to the principle of single variable, 10 healthy females were tested in turns for HSM blood perfusion. The test results showed that the blood flow promotion multiples were better when the sample was perforated and covered, as well as when the width of the covered sample was 10 cm and the time of the covered sample was 20 min.

### 2.4. Measurement of Blood Perfusion in Skin Microcirculation of Magnetic Polypropylene Knitted Fabric

**Figure 2:**
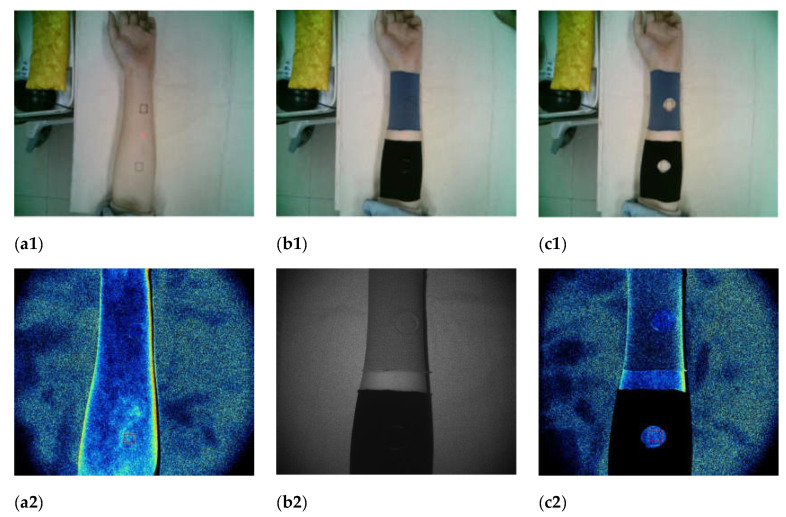
Extended forearm status and blood flow imaging of subjects’ non-dominant hand: (**a1**) after the forearm of the non-dominant hand of the subject is placed on the experimental platform and kept in a relatively stationary state for 15 min, test points 1 and 2 were selected for blood perfusion measurement; (**b1**) covered the sample for 20 min; (**c1**) after the covering sample were removed, test point 1 and test point 2 were selected for blood perfusion measurement; (**a2**) flow imaged when the sample was not covered; (**b2**) covered sample process; (**c2**) blood flow imaged after covering the sample.

Sample size: The width of the sample was 10 cm and the length of test point 1 near the wrist was 19–23 cm. The length of test point 2 near the elbow was 23–27 cm, and Velcro was 4 cm to ensure that the sample was completely wrapped around the forearm and the sample size could be adjusted appropriately. The sample was adopted under the perforated and covered test scheme. When covering the sample, the test point covered a round sample with a diameter of 2.2 cm. The extended forearm status and blood flow imaging of subjects’ non-dominant hand are shown in Figure 2.

### 2.5. Procedures for Blood Perfusion Test

The subject adapted to the laboratory environment according to the above requirements for preparation before the test, and we tried to adjust their physiology to a balanced state;

The BVI Angiograph was turned on and the vertical distance between the instrument host and the forearm test position was adjusted according to the red laser beam emitted by the instrument until the “Invalid” on the computer software page polypropylene appeared as “good”;

Blood perfusion original value tested: Subjects’ non-dominant forearm was placed on the experimental platform for 15 min and remained in a relatively static state. The test software selection function was used according to participants in advance of the test point mark, selected, and test point 1 and 2 values of dynamic perfusion were tested; 100 s of blood perfusion amounted to a relatively stable area of more than 20 s. The test software automatically calculated the average of the blood perfusion in the area;

The blood perfusion test after covering the sample: The steps of the original blood perfusion test were repeated after covering the fabric sample for 20 min, and the average blood perfusion amount after covering the sample at test points 1 and 2 was obtained;

Subjects should sit still for at least 10 min after the end of the tests.

### 2.6. Calculation Method of Blood Flow Promotion Multiple

Using the BVI Angiography test coverage samples before and after the local blood flow perfusion, the cover sample was calculated before and after the blood flow velocity ratio. Then, the blood flow promotion multiple of the covered sample was obtained. The effects of the feeding ratio of MP and GV, and stitch on HSM, were studied. The influences of various factors on HSM were analyzed by an orthogonal experiment, and the horizontal combination of fabric structural parameters with better performance was obtained.

The calculation method of blood flow promotion multiple was based on the laser Doppler flowmetry (LDF) detection method [25]. The calculation steps of blood flow promotion multiple are shown in Equations (1) and (2):

(1) $$F_{}=\frac{f_{1}}{f_{2}}$$

(2) $$N=\frac{F_{h}}{F_{0}}$$

*F* is the blood flow velocity ratio; *F*_0_ is the original blood flow velocity ratio; *F_h_* is the ratio of blood velocity after covering the sample; *f*_1_ is the blood perfusion at test point 1; *f*_2_ is the blood perfusion at test point 2; *N* is the blood flow promotion multiple after covering the sample.

## 3. Results and Discussion

Twelve pieces of seamless knitted fabric were used to test skin–blood microcirculation, and the test results are shown in Table 4.

**Figure 3:**
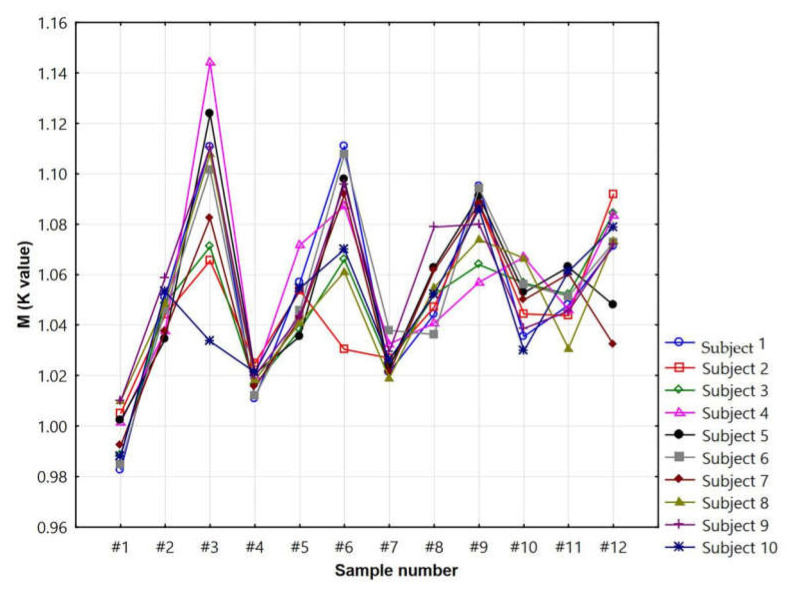
The blood flow promotion multiples of skin–blood microcirculation in the 12 pieces of seamless knitted fabric.

In order to intuitively observe the influence of 12 pieces of seamless knitted fabric on the blood flow promotion multiples of skin–blood microcirculation in 10 healthy females, as well as the test results of the skin–blood microcirculation of the 12 pieces of seamless knitted fabric, a trend chart showing the blood flow promotion multiples of skin–blood microcirculation was made (Figure 3).

Table 4 and Figure 3 showed that samples 1, 2, 4, 5, and 7 had low blood flow promotion multiples. The blood flow promotion multiples of samples 8, 10, 11, and 12 were lower. The blood flow promotion multiples of samples 3, 6, and 9 were higher. Among them, the blood flow promotion multiples of sample 3 were between 1.07 and 1.14. The blood flow promotion multiples of sample 6 were between 1.03 and 1.11. The blood flow promotion multiples of sample 9 were between 1.06 and 1.10. To obtain analysis results more accurately, the average results of 10 healthy females who were tested for blood flow promotion multiples were analyzed. This paper adopts the orthogonal experimental intuitive analysis method to explore the relationship between the skin–blood microcirculation blood flow promotion multiples and the fabric structural parameters of 12 seamless knitted fabrics. The specific results are shown in Table 5.

**Figure 4:**
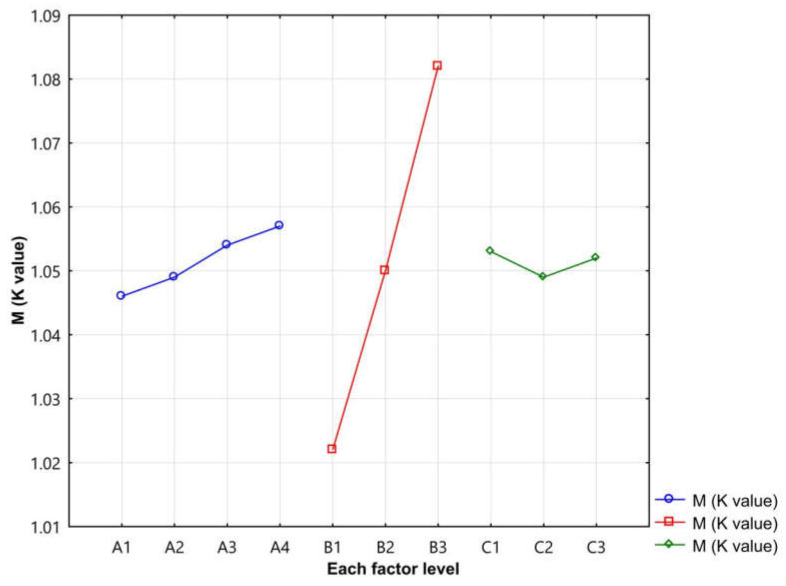
Relationship between skin microcirculation blood flow promotion multiples and fabric structural parameters.

It can be seen from Table 5 that factor B, magnetic powder content of polypropylene, has the greatest influence on skin–blood microcirculation blood flow promotion multiples, followed by factor A, face yarn ratio. Factor C stitch has the least influence. The optimal sample scheme was (A4, B3, C1). When the face yarn ratio was 25:75, the magnetic powder content of the polypropylene was 50%, and the stitch was weft plain stitch. In order to make a more intuitive observation, a relationship between skin microcirculation blood flow promotion multiples and fabric structural parameters was drawn, as shown in Figure 4.

According to the results in Figure 4, in factor B, the order of blood flow promotion multiples sorted was as follows: B3 > B2 > B1, i.e., when the magnetic powder content of polypropylene was 50%, the MP fabric had a better effect on skin–blood microcirculation. When the face yarn ratio was 25:75 and the stitch was weft plain stitch, the blood flow promotion multiples of the skin–blood microcirculation was greater.

## 4. Conclusions

This experiment selected MP yarn and GV yarn as materials and chose three important factors affecting the performance of seamless knitted fabric: the magnetic powder content of polypropylene, the feeding ratio of MP and GV, and stitch. Twelve samples were prepared. References to the LDF detection method and BVI Angiography were made to test the influence of each sample on the HSM of 10 subjects. Finally, the sample with better performance was evaluated by the orthogonal test design with the partial addition method, and the structural parameters of the sample were obtained. The analysis of multiples of skin–blood microcirculation showed the magnetic powder content of polypropylene, followed by the face yarn ratio. Stitch had the least influence. When the face yarn ratio was 100:0, the magnetic powder content of polypropylene was 50%, and the stitch was 1 + 1 false rib, meaning the fabric could promote HSM better than the common polypropylene knitted fabric, in which the blood flow promotion multiples increased by 9.87%. The optimal sample scheme of the face yarn ratio was 25:75, the magnetic powder content of the polypropylene was 50%, and the stitch was weft plain stitch. The purpose of this study was to explore the structural parameters of MP fiber-knitted fabrics that have a better effect on promoting HSM so as to develop textiles that can promote the microcirculation of human skin with excellent performance. Additionally, this study provides a clear reference basis for the application of magnetic health care textiles in the adjuvant treatment of chronic diseases such as scapulohumeral periarthritis.

## Figures and Tables

**Table 1:** Face yarn material and specification.

Number	Face Yarn Material and Specification
1	122.2dtex(110D)MP yarn with 50% magnetic powder content (MP-50)
2	122.2dtex(110D)MP yarn with 10% magnetic powder content (MP-10)
3	122.2dtex(110D)Common polypropylene yarn with 0% magnetic powder content (MP-0)
4	106.3dtex(50s) GV yarn ^1^

^1^ Note: the following: “MP yarn with 50% magnetic powder content (MP-50)” is referred to as “MP-50”; “MP yarn with 10% magnetic powder content (MP-10)” is referred to as“MP-10”; “Common polypropylene yarn with 0% magnetic powder content (MP-0)” is referred to as“MP-0”.

**Table 2:** Fabric factor levels.

Factor Levels	A: Face Yarn Ratio (MP Yarn: GV Yarn)	B: Magnetic Powder Content of Polypropylene	C: Stitch	The Lining Yarn
1	100:0	MP-0	Weft plain stitch	Nylon/spandex-coated yarn
2	75:25	MP-10	1 + 1 false rib	
3	50:50	MP-50	1 + 3 false rib	
4	25:75	-	-	

**Table 3:** Weaving scheme of sample fabric.

Fabric Number	A: Face Yarn Ratio (MP Yarn: GV Yarn)	B: Magnetic Powder Content of Polypropylene	C: stitch
#1	100:0	MP-0	Weft plain stitch
#2	100:0	MP-10	1 + 1 false rib
#3	100:0	MP-50	1 + 3 false rib
#4	75:25	MP-0	1 + 1 false rib
#5	75:25	MP-10	1 + 3 false rib
#6	75:25	MP-50	Weft plain stitch
#7	50:50	MP-0	1 + 3 false rib
#8	50:50	MP-10	Weft plain stitch
#9	50:50	MP-50	1 + 1 false rib
#10	MP:GV = 25:75	MP-0	Weft plain stitch
#11	MP:GV = 25:75	MP-10	1 + 1 false rib
#12	MP:GV = 25:75	MP-50	1 + 3 false rib

**Table 4:** The blood flow promotion multiples of skin–blood microcirculation.

Sample Number	The Subjects											
	Subject 1	Subject 2	Subject 3	Subject 4	Subject 5	Subject 6	Subject 7	Subject 8	Subject 9	Subject 10	The Average	Root Mean Square
#1	0.9825	1.0049	0.9886	1.0015	1.0023	0.9848	0.9923	1.0098	1.0099	0.9879	0.9964	0.9965
#2	1.0509	1.0441	1.0484	1.0376	1.0343	1.0459	1.0374	1.0485	1.0586	1.0532	1.0459	1.0459
#3	1.1106	1.0655	1.0710	1.1441	1.1238	1.1016	1.0823	1.1079	1.1104	1.0336	1.0951	1.0955
#4	1.0108	1.0248	1.0162	1.0199	1.0208	1.0120	1.0155	1.0183	1.0199	1.0214	1.0180	1.0180
#5	1.0567	1.0535	1.0382	1.0717	1.0355	1.0458	1.0427	1.0409	1.0435	1.0546	1.0483	1.0484
#6	1.1108	1.0303	1.0660	1.0873	1.0977	1.1078	1.0917	1.0610	1.0958	1.0700	1.0818	1.0821
#7	1.0214	1.0269	1.0241	1.0325	1.0233	1.0378	1.0212	1.0190	1.0294	1.0263	1.0262	1.0262
#8	1.0442	1.0470	1.0520	1.0407	1.0626	1.0362	1.0616	1.0548	1.0789	1.0522	1.0530	1.0531
#9	1.0950	1.0876	1.0639	1.0570	1.0911	1.0943	1.0881	1.0739	1.0800	1.0858	1.0817	1.0817
#10	1.0354	1.0444	1.0566	1.0669	1.0528	1.0561	1.0499	1.0664	1.0387	1.0298	1.0497	1.0498
#11	1.0478	1.0437	1.0522	1.0459	1.0629	1.0513	1.0603	1.0307	1.0455	1.0612	1.0501	1.0502
#12	1.0711	1.0917	1.0843	1.0835	1.0478	1.0727	1.0323	1.0733	1.0719	1.0787	1.0707	1.0709

**Table 5:** Orthogonal analysis table of blood flow promotion multiples of skin–blood microcirculation.

Sample Number	Factors			Multiple of Blood Flow Promotion (M)
	A	B	C	
#1	1	1	1	0.9964
#2	1	2	2	1.0459
#3	1	3	3	1.0951
#4	2	1	2	1.0180
#5	2	2	3	1.0483
#6	2	3	1	1.0818
#7	3	1	3	1.0262
#8	3	2	1	1.0530
#9	3	3	2	1.0817
#10	4	1	1	1.0497
#11	4	2	2	1.0501
#12	4	3	3	1.0707
K_1_	1.046	1.022	1.053	-
K_2_	1.049	1.050	1.049	-
K_3_	1.054	1.082	1.052	-
K_4_	1.057	-	-	-
R	0.011	0.060	0.003	-
Optimal level	A4	B3	C1	-

## Data Availability

The data presented in this study are available on request from the corresponding author. The data are not publicly available due to a complicated structure that requires additional explanations.
